# Association of the fibronectin type III domain–containing protein 5 rs1746661 single nucleotide polymorphism with reduced brain glucose metabolism in elderly humans

**DOI:** 10.1093/braincomms/fcad216

**Published:** 2023-08-17

**Authors:** Ricardo A S Lima-Filho, Andréa L Benedet, Marco Antônio De Bastiani, Guilherme Povala, Danielle Cozachenco, Sergio T Ferreira, Fernanda G De Felice, Pedro Rosa-Neto, Eduardo R Zimmer, Mychael V Lourenco, Alzheimer’s Disease Neuroimaging Initiative

**Affiliations:** Institute of Medical Biochemistry Leopoldo de Meis, Federal University of Rio de Janeiro, Rio de Janeiro, RJ 21941-902, Brazil; Department of Psychiatry and Neurochemistry, The Sahlgrenska Academy at the University of Gothenburg, Mölndal, 413 45, Sweden; Graduate Program in Biological Sciences: Pharmacology and Therapeutics, Institute of Basic Health Sciences, Federal University of Rio Grande do Sul, Porto Alegre, 90035-003, Brazil; Graduate Program in Biological Sciences: Pharmacology and Therapeutics, Institute of Basic Health Sciences, Federal University of Rio Grande do Sul, Porto Alegre, 90035-003, Brazil; Institute of Medical Biochemistry Leopoldo de Meis, Federal University of Rio de Janeiro, Rio de Janeiro, RJ 21941-902, Brazil; Institute of Medical Biochemistry Leopoldo de Meis, Federal University of Rio de Janeiro, Rio de Janeiro, RJ 21941-902, Brazil; Institute of Biophysics Carlos Chagas Filho, Federal University of Rio de Janeiro, Rio de Janeiro, RJ 21941-902, Brazil; D’Or Institute for Research and Education (IDOR), Rio de Janeiro, RJ 22281-100, Brazil; Institute of Medical Biochemistry Leopoldo de Meis, Federal University of Rio de Janeiro, Rio de Janeiro, RJ 21941-902, Brazil; D’Or Institute for Research and Education (IDOR), Rio de Janeiro, RJ 22281-100, Brazil; Centre for Neuroscience Studies, Department of Biomedical and Molecular Sciences & Department of Psychiatry, Queen’s University, Kingston, ON K7L 3N6, Canada; Translational Neuroimaging Laboratory (TNL), McGill Center for Studies in Aging (MCSA), Douglas Mental Health University Institute, Departments of Neurology and Neurosurgery, Psychiatry, and Pharmacology, McGill University, Montreal, QC H4H 1R3, Canada; Graduate Program in Biological Sciences: Pharmacology and Therapeutics, Institute of Basic Health Sciences, Federal University of Rio Grande do Sul, Porto Alegre, 90035-003, Brazil; Department of Pharmacology, Universidade Federal do Rio Grande do Sul, Porto Alegre, 90035-003, Brazil; Institute of Medical Biochemistry Leopoldo de Meis, Federal University of Rio de Janeiro, Rio de Janeiro, RJ 21941-902, Brazil

**Keywords:** Alzheimer’s disease, FNDC5/irisin, PET-FDG, glucose metabolism, single nucleotide polymorphism

## Abstract

Fibronectin type III domain–containing protein 5 (FNDC5) and its derived hormone, irisin, have been associated with metabolic control in humans, with described FNDC5 single nucleotide polymorphisms being linked to obesity and metabolic syndrome. Decreased brain FNDC5/irisin has been reported in subjects with dementia due to Alzheimer’s disease. Since impaired brain glucose metabolism develops in ageing and is prominent in Alzheimer’s disease, here, we examined associations of a single nucleotide polymorphism in the FNDC5 gene (rs1746661) with brain glucose metabolism and amyloid-β deposition in a cohort of 240 cognitively unimpaired and 485 cognitively impaired elderly individuals from the Alzheimer’s Disease Neuroimaging Initiative. In cognitively unimpaired elderly individuals harbouring the FNDC5 rs1746661(T) allele, we observed a regional reduction in low glucose metabolism in memory-linked brain regions and increased brain amyloid-β PET load. No differences in cognition or levels of cerebrospinal fluid amyloid-β_42_, phosphorylated tau and total tau were observed between FNDC5 rs1746661(T) allele carriers and non-carriers. Our results indicate that a genetic variant of FNDC5 is associated with low brain glucose metabolism in elderly individuals and suggest that FNDC5 may participate in the regulation of brain metabolism in brain regions vulnerable to Alzheimer’s disease pathophysiology. Understanding the associations between genetic variants in metabolism-linked genes and metabolic brain signatures may contribute to elucidating genetic modulators of brain metabolism in humans.

## Teaser

Lima-Filho *et al.* reported that cognitively unimpaired elders carrying the *FNDC5* rs1746661(T) allele develop low brain glucose metabolism and increased amyloid deposition. These findings indicate that *FNDC5* may contribute to regional glucose metabolism in the human brain.

## Graphical Abstract

**Graphical Abstract:**
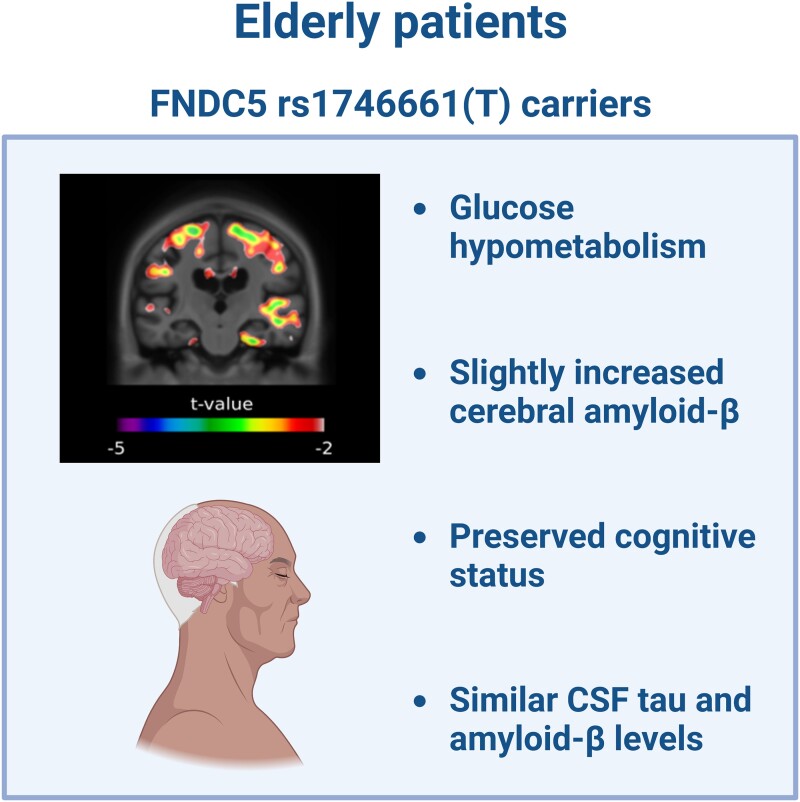

## Introduction

Irisin is an exercise-induced myokine that originates from proteolytic cleavage of fibronectin type III domain–containing protein 5 (FNDC5), a transmembrane protein expressed in several tissues, including the skeletal muscle and brain.^1,2^ Irisin was initially reported to reprogramme adipocyte metabolism and control peripheral glucose homeostasis.^3,4^*FNDC5* single nucleotide polymorphisms (SNPs) have been associated with obesity and metabolic syndrome in humans.^5‐7^

In the brain, FNDC5/irisin induces the expression of neurotrophins and synaptic plasticity-related genes^2,8^ and mediates, at least in part, exercise-induced neurogenesis in the mouse hippocampus.^9^ FNDC5/irisin expression is reduced in the prefrontal cortex and cerebrospinal fluid (CSF) of patients diagnosed with major depression^10,11^ and has also been linked to Parkinson’s disease.^12,13^ We and others have shown that FNDC5/irisin is reduced in the Alzheimer’s disease (AD) brain and CSF and that replenishing its levels rescues pathology and memory in mouse models of AD.^1,9^ We also reported that CSF irisin correlates with CSF levels of amyloid-β_42_ (Aβ_42_) and brain-derived neurotrophic factor and cognition in humans.^11,14^ However, whether there are genomic associations between *FNDC5* and AD-related biomarkers remains unknown.

AD has been linked to defective brain hormonal signalling and energy metabolism.^15‐19^ Consolidated evidence indicates that AD brains develop hypometabolic profiles in humans.^18,20‐23^ However, the underlying causes and risk factors for this association remain to be fully determined. Investigation of brain glucose metabolism using [^18^F]fluorodeoxyglucose positron emission tomography (FDG-PET) is a powerful tool to identify and study brain metabolic dysfunction in ageing and age-related diseases.^24,25^

Here, we hypothesized that polymorphisms in the *FNDC5* gene may affect brain function and AD biomarkers in the elderly. We thus analysed data from 240 cognitively unimpaired (CU) and 485 cognitively impaired (CI) participants from the Alzheimer’s Disease Neuroimaging Initiative (ADNI) cohort to investigate if SNPs within the *FNDC5* gene are associated with changes in FDG-PET, cognition and AD biomarkers. Allele-specific changes associated with the *FNDC5* gene may shed light on the roles of FNDC5/irisin in brain physiology and neurodegeneration.

## Materials and methods

### Study design and ethics

Data used to prepare this article were obtained from the ADNI database (adni.loni.usc.edu). The ADNI was launched in 2003 as a public–private partnership led by Principal Investigator Michael W. Weiner, MD. The primary goal of ADNI has been to test whether serial MRI, PET, other biological markers and clinical and neuropsychological assessment can be combined to measure the progression of mild cognitive impairment and early AD. The ADNI studies obtained appropriate approvals from institutional review boards. Access to data was preapproved by the ADNI Review Board.

The current study comprised 725 elderly individuals with available cross-sectional [^18^F]FDG-PET and genotyping data: 240 CU (*n* = 82 carriers and 158 non-carriers) and 485 CI individuals (*n* = 167 carriers and 318 non-carriers). Patients in the CI group were clinically diagnosed with either AD-linked dementia (*n* = 66) or mild cognitive impairment (*n* = 419). For more detailed demographics, see Table 1.

**Table 1:** Cohort demographics and diagnostic information

	CU	CI	Overall
	240	485	725
Age (years)	75.5 (6.25)	72.9 (7.73)	73.8 (7.37)
Sex (F)	123 (51.3%)	208 (42.9%)	331 (45.7%)
Education (years)	16.4 (2.69)	16.0 (2.74)	16.2 (2.73)
**Cognitive status**			
Alzheimer’s disease	0 (0%)	66 (13.6%)	66 (9.1%)
Mild cognitive impairment	0 (0%)	419 (86.4%)	419 (57.8%)
Cognitively unimpaired	240 (100%)	0 (0%)	240 (33.1%)
**Florbetapir (positivity)**			
Negative	120 (50.0%)	163 (33.6%)	283 (39.0%)
Positive	65 (27.1%)	233 (48.0%)	298 (41.1%)
**Florbetapir (SUVR)**	1.11 (0.181)	1.23 (0.231)	1.19 (0.222)
**FNDC5 rs1746661(T) SNP**			
Carriers	74 (30.8%)	146 (30.1%)	220 (30.3%)
Non-carriers	166 (69.2%)	339 (69.9%)	505 (69.7%)
**ApoE-ε4**			
Carriers	64 (26.7%)	232 (47.8%)	296 (40.8%)
Non-carriers	176 (73.3%)	253 (52.2%)	429 (59.2%)

Age, sex, diagnosis, educational status, florbetapir positivity and SUVR values, FNDC5 rs1746661(T) SNP frequency and ApoE-ε4 status are indicated as mean with SD or counts with percentage of total (%). Abbreviations: ApoE-ε4 = apolipoprotein E allele ε4; CI = cognitively impaired; CU = cognitively unimpaired; FNDC5 = fibronectin type III domain–containing protein 5; IQR = interquartile range; SNP = single nucleotide polymorphism; SD = standard deviation; SUVR = standardized uptake value ratio.

### PET imaging

PET acquisitions followed the protocols established by ADNI (http://adni.loni.usc.edu/methods). [^18^F]FDG-PET images were preprocessed to produce an effective point spread function of full-width at half maximum of 8 mm. Subsequently, linear registration and nonlinear normalization to the MNI152 template were performed with the linear and nonlinear transformations derived from the automatic PET to MRI transformation and the individual’s anatomical MRI coregistration. [^18^F]FDG-PET standardized uptake value ratio (SUVR) maps were generated using pons as the reference region.^26,27^ The global average SUVR was obtained from ADNI. Additional details on our processing pipeline can be found elsewhere^28,29^ (see also https://adni.loni.usc.edu/wp-content/uploads/2010/05/ADNIGO_PET_Tech_Manual_01142011.pdf). Aβ load was estimated using [^18^F]florbetapir, and the SUVR was calculated using the cerebellar grey matter as the reference region. The global amyloid PET average SUVR was obtained from ADNI, and the cut-off for positivity is 1.11 SUVR.

### CSF measurements

CSF was collected from donors by lumbar puncture following ADNI protocols and analysed by the electrochemiluminescence immunoassay Elecsys Aβ_42_, phosphorylated tau at threonine 181 (p-Tau_181_) and total tau (t-Tau) on an automated Elecsys Cobas e601 instrument, as described by ADNI (https://adni.loni.usc.edu/methods/documents/). Precision and accuracy of runs were assessed and were within the stated limits set by the manufacturer.^30,31^ The following thresholds were applied to determine amyloid or tau positivity: Aβ_42_ < 680 pg/ml [defined as amyloid-positive (A+)]^32^ and t-Tau > 266 pg/ml [defined as tau-positive (T+)].^33^

### Genetic analysis

PLINK (v1.9) was used to preprocess genetic data and SNP selection. Participants were genotyped with the HumanOmni2.5 BeadChip array (Illumina, Inc., San Diego, CA, USA). Quality control was performed by excluding SNPs with a genotyping efficiency of <95%, a minor allele (MA) frequency of <5%, or a deviation from the Hardy–Weinberg equilibrium of <1 × 10^−6^. Subjects would be excluded if they had a call rate of <95% or if genetic relatedness was detected (PI_HAT > 0.5). Redundant SNPs in high linkage disequilibrium were removed based on pairwise correlation (*R*^2^ = 0.8). After the initial processing of the genetic data, SNPs from the *FNDC5* gene were extracted within a 10 kb window upstream and downstream of the gene. We followed the STREGA guidelines,^34^ as noted in the accompanying checklist.

### Statistical analysis

Statistical analysis was performed using GraphPad Prism 8 (GraphPad Software Inc., La Jolla, CA, USA). Data were checked for normal distribution using the D’Agostino & Pearson Omnibus normality test. Statistical significance of differences between average values of carriers and non-carriers was assessed using a two-way ANOVA test, followed by Tukey’s posttest, unless otherwise stated in legends. In the supplementary figures, where carriers were subdivided between homozygous and heterozygous, a two-tailed one-way ANOVA test, followed by Dunnett’s *post hoc* test, was performed. *P*-values are reported in each graph.

For [^18^F]FDG-PET imaging analysis, voxel-wise analyses were performed using Rminc, where linear models tested the association between the *FNDC5* SNP carriership and [^18^F]FDG-PET in all participants, adjusting for age, sex and diagnosis. In addition, the analysis was repeated within CU and CI groups, adjusting for covariates. Adjustment for multiple comparisons was done with random field theory,^35^ and significant *t*-values are below or equal to −3.1.

## Results

### SNP screening and population characteristics

After the initial screening of the genomic region of human chromosome 1 containing the *FNDC5* locus (±10 kb), five SNPs were extracted (rs11580896, rs6673337, rs12126851, kgp10883921 and rs1746661). In this study, we chose to focus on rs1746661(G/T), as it was the only SNP located within the *FNDC5* locus.

Demographics and total *FNDC5* transcript counts are presented in Table 1 and Supplementary Table 1. There were no significant differences in age, sex, apolipoprotein E allele ε4 (ApoE-ε4) status or frequency of cognitive impairment and AD between carrier and non-carrier groups (Supplementary Table 1). *FNDC5* transcript counts were similar between carrier and non-carrier groups (Supplementary Table 1), indicating no alterations in *FNDC5* mRNA content between groups.

### Association between FNDC5 and [^18^F]FDG-PET in humans

**Figure 1:**
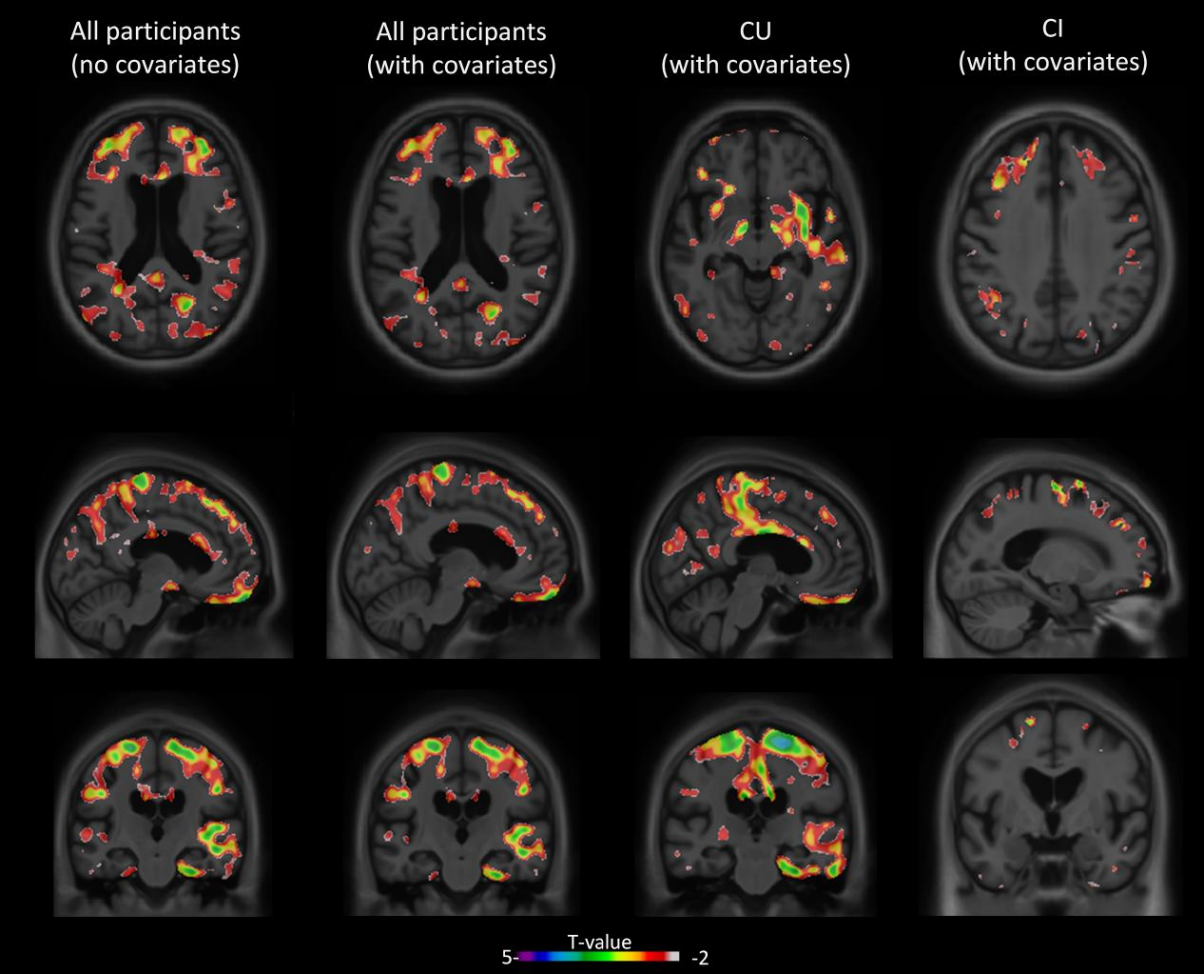
**Carriers of the *FNDC5* rs1746661(T) SNP present brain glucose hypometabolism.** Figure shows transversal (upper row), sagittal (middle row) and coronal (lower row) images showing FDG-PET metabolism in *FNDC5* rs1746661(T) carriers compared to non-carriers in all participants without adjustments for covariates (left) and with adjustments for covariates (age and sex) (centre left). Similarly, in CU participants’ *FNDC5* rs1746661(T) carriers had reduced FDG-PET metabolism as compared to non-carriers (adjusting for covariates; centre right). CI participants’ *FNDC5* rs1746661(T) carriers also had reduced FDG-PET metabolism as compared to non-carriers (adjusting for covariates; right) but to a much lesser extent than that observed in CU participants. *t*-values are shown as a colour scale in each image, and significant results have a *t*-value smaller than or equal to −3.1.

**Figure 2:**
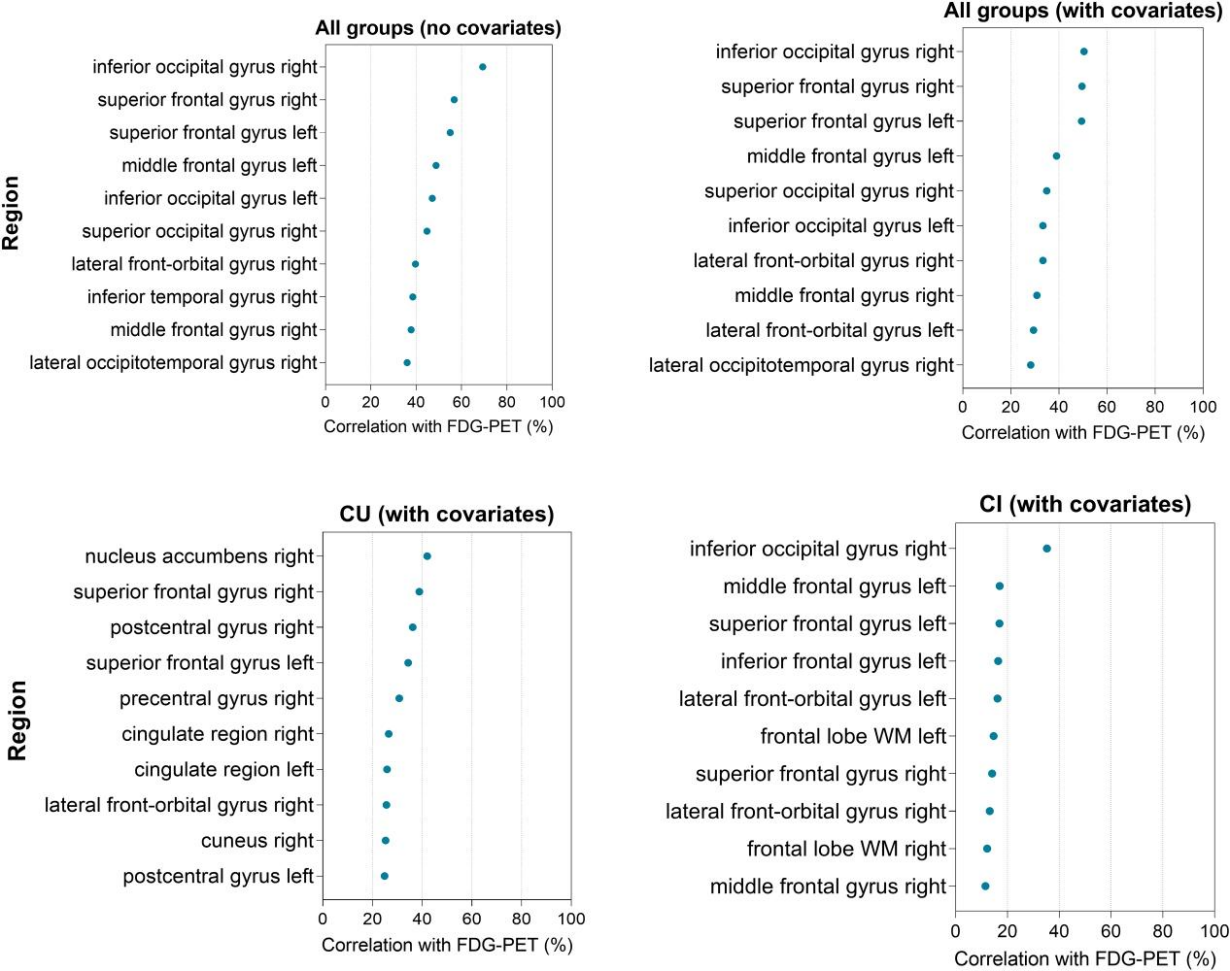
**Regional correlation percentage of FDG-PET signals with the rs1746661(T) allele.** Figure shows brain regions ranked by the percentage of significant voxels in the grey matter, determined through voxel-wise analysis between [^18^F]FDG-PET and FNDC5 using a *t*-statistical map (threshold of *t*-value = 2) from the generalized linear regression model.

We investigated if rs1746661 MA carriers (T) presented alterations in brain glucose metabolism through regional [^18^F]FDG-PET in the brain. In the overall cohort, we found that CU MA carriers presented [^18^F]FDG-PET hypometabolism compared to non-carriers in multiple brain areas (Fig. 1). Analyses of significant [^18^F]FDG-PET voxels in the grey matter across brain regions revealed that impaired glucose metabolism was more prominently observed in brain areas linked to cognition, executive function and spatial processing, including the superior frontal gyrus and the inferior occipital gyrus in the overall cohort after covariate adjustment for sex and age (Figs 1 and 2). Notably, additional hypometabolic areas (nucleus accumbens, postcentral gyrus and parietal lobe white matter tracts) emerged in CU rs1746661 MA carriers compared to non-carriers (Figs 1 and 2; Supplementary Fig. 1A), suggesting that rs1746661(T) is associated with signs of defective brain glucose metabolism in subjects without cognitive impairment. A complete list of investigated regions and their voxel association with the rs1746661 MA in CU individuals is presented in Supplementary Fig. 1. Conversely, only small clusters showing subtle differences between genotypes were found in CI subjects (Figs 1 and 2).

### FNDC5, AD biomarkers and cognitive performance

**Figure 3:**
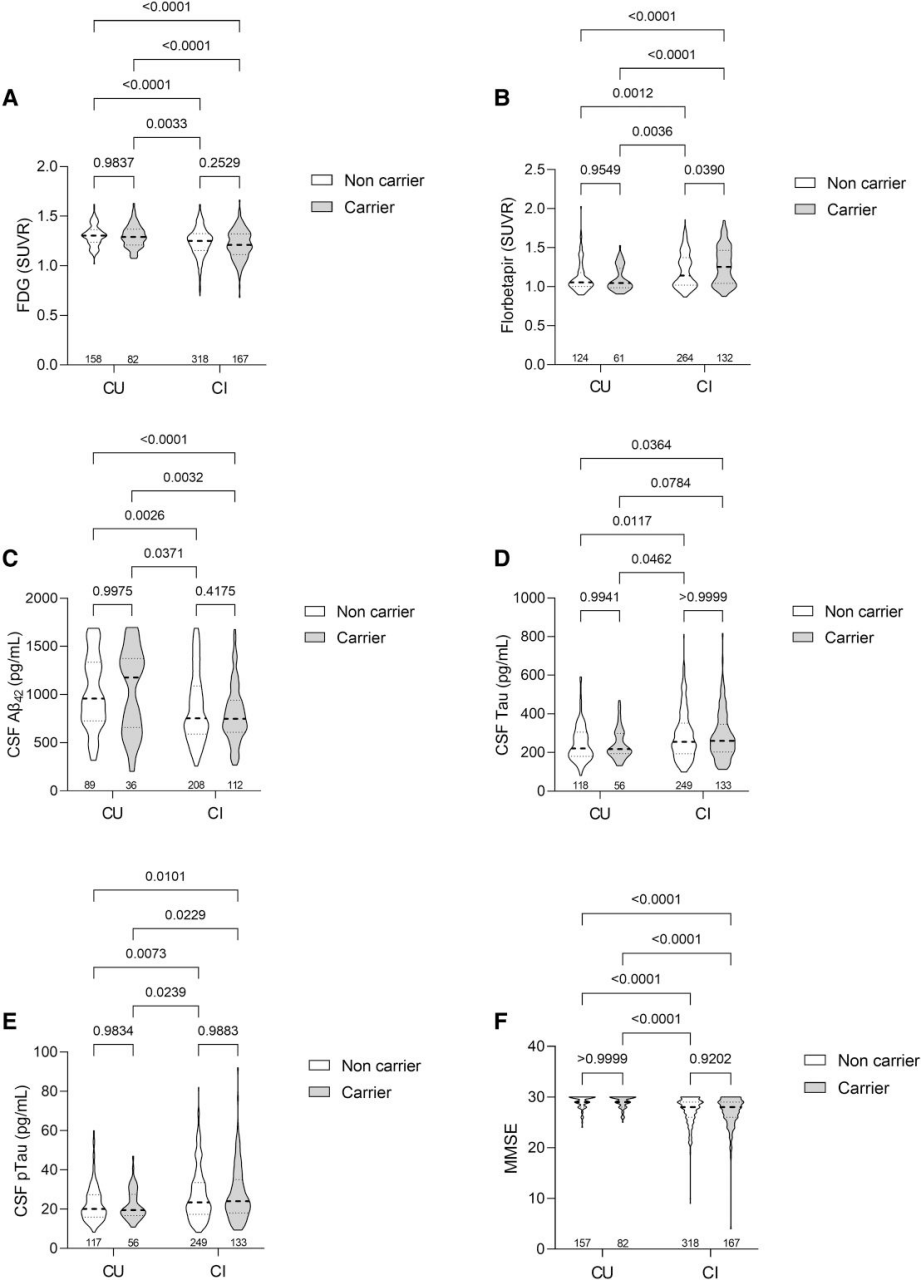
**Brain amyloid PET, CSF biomarkers and cognition in CU and CI *FNDC5* rs1746661(T) carriers and non-carriers.** FDG-PET SUVR [interaction: *F*(1,721) = 0.5834; *P* = 0.4452; cog. status: *F*(1,721) = 42.19; *P* < 0.0001; SNP: *F*(1,721) = 1.842; *P* = 0.1751] (**A**); florbetapir (amyloid) PET SUVR [interaction: *F*(1,577) = 3.728; *P* = 0.0540; cog. status: *F*(1,577) = 38.80; *P* < 0.0001; SNP: *F*(1,577) = 1.157; *P* = 0.2825] (**B**); CSF Aβ_42_ [interaction: *F*(1,441) = 0.9008; *P* = 0.3431; cog. status: *F*(1,441) = 23.49; *P* < 0.0001; SNP: *F*(1,441) = 0.3811; *P* = 0.5373] (**C**); tau [interaction: *F*(1,552) = 0.03853; *P* = 0.8445; cog. status: *F*(1,552) = 13,93; *P* = 0.0002; SNP: *F*(1,552) = 0.05279; *P* = 0.8184] (**D**); p-Tau [interaction: *F*(1,551) = 0.2329; *P* = 0.6296; cog. status: *F*(1,551) = 17.57; *P* < 0.0001; SNP: *F*(1,551) = 0.01584; *P* = 0.8999] (**E**); and Mini-Mental State Exam scores [interaction: *F*(1,720) = 0.1326; *P* = 0.7158; cog. status: *F*(1,720) = 77.58; *P* < 0.0001; SNP: *F*(1,720) = 0.1345; *P* = 0.7140] (**F**) in CU and CI non-carriers and carriers of the *FNDC5* rs1746661(T) SNP. Adjusted *P*-values are depicted above bars, and sample size for each group is depicted below bars. Two-way ANOVA with Tukey’s multiple comparison was performed.

We then investigated whether rs1746661 MA carriers presented alterations in AD biomarkers. As expected, a reduced global [^18^F]FDG-PET signal was observed in CI patients when compared to CU patients, but we found no differences between genotype groups (Fig. 3A), suggesting that the changes in the [^18^F]FDG-PET signal in CU SNP carriers are present in specific brain regions rather than widespread glucose hypometabolism. We then investigated PET data to assess cerebral Aβ load. We found increased [^18^F]florbetapir PET retention in CI subjects compared to CU individuals, indicating higher Aβ deposition (Fig. 3B). Of note, CI rs1746661(T) carriers had significantly higher Aβ PET SUVR than CI non-carriers (Fig. 3B), indicating an association between the SNP and Aβ accumulation. CSF levels of Aβ_42_, t-Tau or p-Tau_181_ between carriers and non-carriers were unchanged, although differences between CU and CI were significant (Fig. 3C–E). In addition, the proportion of subjects with abnormal CSF Aβ_42_ (<680 pg/ml; defined as A+)^32^ or t-Tau (>266 pg/ml; defined as T+)^33^ was similar between carriers and non-carriers (Supplementary Table 1). Similar findings were observed in [^18^F]florbetapir retention when comparisons were performed between carriers and non-carriers in either CU or CI (Supplementary Table 2). When MA carriers were stratified by the allele copy number, CSF Aβ_42_ was unchanged (Supplementary Fig. 2A), but homozygous individuals exhibited a trend for increased CSF t-Tau (Supplementary Fig. 2B) and p-Tau_181_ (Supplementary Fig. 2C), though the limited representation of these individuals in the studied cohort reduced the statistical power for this analysis. Mini-Mental State Exam scores were lower in the CI group but similar between MA carriers and non-carriers in either group (Fig. 3E).

## Discussion

Irisin has been shown to mediate brain benefits of physical exercise,^1,2,9^ to rescue AD-linked phenotypes in mouse models^1^ and to correlate with Aβ_42_ and cognitive impairment in AD.^14,36^ However, insight into how FNDC5 and irisin contribute to brain physiology and disease risk is limited. Here, we studied the influence of a SNP found in an intronic region of the *FNDC5* locus (rs1746661) on AD biomarkers and FDG-PET in a cohort of human subjects with or without cognitive impairment.

Irisin has been shown to modulate key peripheral^3,37^ and brain^1,2,9^ pathways linked to metabolism.^38,39^ Our finding that rs1746661(T) carriers exhibit FDG hypometabolism in brain regions important for cognition advocates for the potential roles of this *FNDC5* genomic variant in the control of brain metabolism and function.

The functional relevance of rs1746661 to brain metabolism needs to be further investigated. While previous reports found little evidence of diabetes-linked peripheral metabolic changes associated with rs1746661 in humans,^5,6^ rs1746661(T) has been associated with increased blood pressure and imbalanced plasma cholesterol and triglyceride levels^6,40^ in type 2 diabetes patients. Since *FNDC5* is notably expressed in the brain and in peripheral tissues,^1,2^ additional investigation should aim to establish whether impaired brain metabolism in SNP carriers originates from brain or peripheral metabolic alterations.

Accumulating evidence indicates that intronic SNPs play key roles in regulating transcription.^41‐43^ Here, the carriership of rs1746661(T) did not result in changes in the total number of *FNDC5* transcripts (Supplementary Table 1), suggesting this SNP may impact other transcriptional characteristics (e.g. splicing) rather than transcription efficiency. Notably, the rs1746661 SNP is located in a relevant intergenic region (between Exons 4 and 5). *FNDC5* Exon 4 codes for amino acids that are part of the cleaved peptide termed irisin,^40^ while Exon 5 has been shown as an important source of transcript variation for *FNDC5.*^44^ Future functional genomics studies are warranted to determine the significance of rs1746661(T) in brain metabolism and AD pathological features.

We focused on potential associations of the rs1746661 SNP with cognitive function, Aβ or tau pathology, as they are hallmarks of AD pathology and disease progression.^45^ No significant difference in the prevalence of cognitive impairment or AD was detected between carriers and non-carriers of rs1746661 MA. However, brain Aβ deposition was increased in carriers, even when compared with CI non-carriers, and a trend towards CSF tau build-up was observed in subjects carrying two copies of rs1746661(T), though a larger cohort is necessary to confirm this observation.

Our results also show a more prominent regional low glucose metabolism in CU subjects, while results from the CI cohort denote more subtle changes, which may be explained by the fact that CI subjects already present a larger, global low glucose metabolism in the brain. It is possible that the extent of brain hypometabolism found in SNP carriers may contribute to preclinical memory defects at very early phases of disease progression. This possibility could be explored in longitudinal studies with SNP carriers in the future. The interaction of *FNDC5* (rs1746661) with known genetic risk factors for sporadic AD, such as ApoE-ε4 and triggering receptor expressed on myeloid cells 2, to predispose the brain to neurodegenerative changes should also be investigated.

Limitations of the current study include the reduced size of the group of homozygous *FNDC5* SNP rs1746661(T), which prevented further stratification in our analysis. Moreover, the use of cross-sectional data limits our exploratory potential to predict disease progression in this cohort, and we feel that longitudinal studies of disease progression in carriers versus non-carriers are warranted.

We acknowledge that altered blood perfusion may impact FDG-PET signals. This issue has not been considered in our work because the ADNI study has strict inclusion/exclusion criteria in which the subject must not have other neurological conditions and no signs of previous cerebrovascular disorders, pacemakers, aneurysm clips or artificial heart valves. The subjects must also have good general health and show no local lesions or infarctions in a baseline MRI scan. Therefore, subjects in this cohort are unlikely to present conditions that can impact their cerebral blood perfusion.

To the best of our knowledge, this is the first report to associate an *FNDC5* SNP with low brain glucose metabolism and AD biomarkers. The finding that an *FNDC5* SNP is associated with regional low brain glucose metabolism in humans encourages future studies in larger cohorts to replicate and extend these observations. Further, it may also stimulate investigation of the genomic control and physiological roles of FNDC5/irisin in the brain and how they may interact with the pathophysiology of AD.

## Supplementary Material

fcad216_Supplementary_Data

## Data Availability

All data generated or analysed during the study are included in this article. Raw data are available from the corresponding authors upon reasonable request.
