# Supplementary material for: Association of the fibronectin type III domain–containing protein 5 rs1746661 single nucleotide polymorphism with reduced brain glucose metabolism in elderly humans
Source: Brain Commun. 2023 Aug 17;5(4):fcad216. doi: 10.1093/braincomms/fcad216 (PMC10438215; doi:10.1093/braincomms/fcad216)
Supplement: fcad216_Supplementary_Data [file fcad216_supplementary_data.doc]

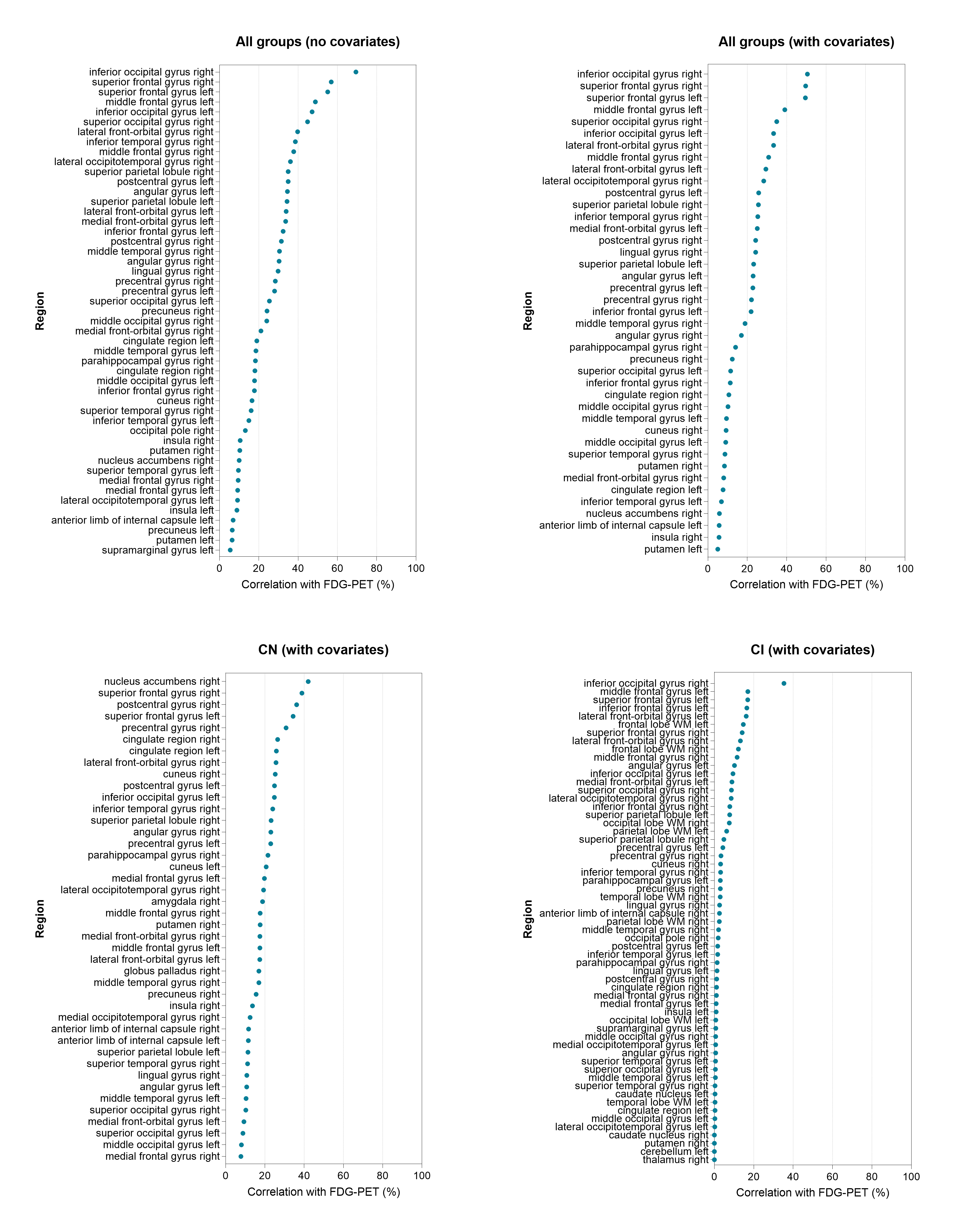
**Supplementary Figures**

**Supplementary Figure 1. Regional correlation percentage with the rs1746661(T) SNP.** The percentage of voxels for each region correlated with FDG-PET are represented from general linear models using all groups with no adjustment for covariates (top left), all groups with covariates adjusted (top right), and CU and CI with covariates adjusted (bottom left and right). WM: white matter.


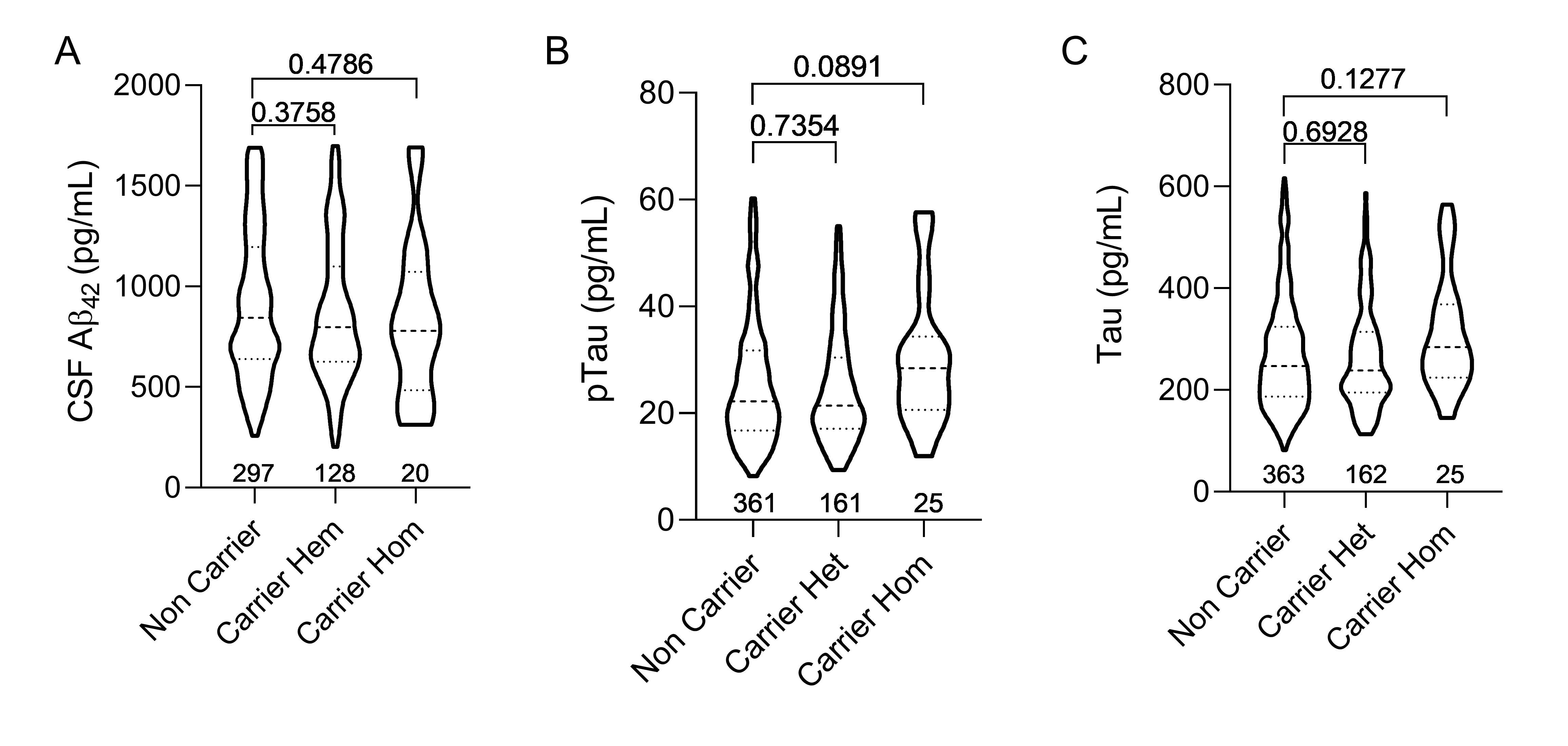


**Supplementary Figure 2. Levels of CSF Aβ_42_, Tau and pTau stratified by copy number**. CSF amyloid-β_42_ (A) [F(2,442) = 1.205; P=0.3006], p-Tau (B) [F(2,544) = 2.490; P=0.0838], Tau (C) [F(2,547) = 2.221; P=0.1095] in non-carriers, heterozygous and homozygous carriers of the *FNDC5* rs1746661(T) allele. p-values are depicted above bars and sample size for each group is depicted below bars. One-way ANOVA with Dunnett’s pos-hoc test.

**Supplementary Table 1: Demographics and diagnostic information as denoted by SNP carriership.**

| **FNDC5 rs1746661 SNP** | **Noncarriers (G)** | **Carriers (T)** | **p-value** |
| --- | --- | --- | --- |
| **Sample size** | 476 | 249 (220 +/-; 29 +/+) | - |
| **Age (years)** | 73,6 ± 7,6 (55,1 - 93,8) | 74,13 ± 6,9 (56,6 - 89,3) | 0,3812 |
| **Sex, % female** | 46,43 | 44,18 | 0,5632 |
| **CI, % positive** | 58,19 | 57,03 | 0,9425 |
| **AD, % positive** | 8,61 | 10,04 | 0,5754 |
| **CSF Aβ+ (%)** | 29.29 | 37.16 | 0,1056 |
| **CSF Tau+ (%)** | 57,18 | 56,68 | 0,9111 |
| ***FNDC5* transcript count** | 8224 ± 407,4 (7382 - 11646) | 8254 ± 420,1 (7115 - 9713) | 0,3045 |

Age and FNDC5 transcript count are indicated as mean ± SD (range). Statistical significances of proportions were analyzed using the chi-square test. Age was analyzed by two-tailed Student’s t-test and total *FNDC5* transcript count were analyzed by Mann Whitney, as distributions were not Gaussian.

Abbreviations: Aβ, amyloid-β; AD, Alzheimer's disease; CI, cognitively impaired subjects; CSF, cerebrospinal fluid; FNDC5, fibronectin type III domain-containing protein 5.

**Supplementary Table 2: Amyloid positivity (by Florbetapir) by cognitive status and SNP carriership.**

| **CU** | **Carrier** | **Non-carrier** | **Overall** |
| --- | --- | --- | --- |
| **Florbetapir** |  |  |  |
| **Sample size** | 74 | 166 | 240 |
| Aβ- | 33 (44.6%) | 87 (52.4%) | 120 (50.0%) |
| Aβ+ | 20 (27.0%) | 45 (27.1%) | 65 (27.1%) |
| Missing | 21 (28.4%) | 34 (20.5%) | 55 (22.9%) |
| **CI** | **Carrier** | **Non-carrier** | **Overall** |
| **Florbetapir** |  |  |  |
| **Sample size** | 146 | 339 | 485 |
| Aβ- | 39 (26.7%) | 124 (36.6%) | 163 (33.6%) |
| Aβ+ | 74 (50.7%) | 159 (46.9%) | 233 (48.0%) |
| Missing | 33 (22.6%) | 56 (16.5%) | 89 (18.4%) |

Proportion of amyloid positive, amyloid negative and missing data for subjects divided by cognitive status and FNDC5 rs1746661 SNP carriership. Statistical significances were analyzed using the chi-square test. Within CU, there was no difference in amyloid positivity/negativity between genotypes (χ^2^ = 0.08, p = 0.76). No significant difference was observed within CI participants (χ^2^ = 2.54, p = 0.11). Thus, amyloid positive participants were equally distributed across genotype groups when all participants were evaluated (χ^2^ = 2.35, p = 0.13).

Abbreviations: Aβ, amyloid-β; AD, Alzheimer's disease; CI, cognitively impaired subjects; CU, cognitively unimpaired; FNDC5, fibronectin type III domain-containing protein 5.
